# Friendship or Competition? Symmetry in Social Play within the Two Packs of German Shepherd Puppies

**DOI:** 10.3390/ani10091627

**Published:** 2020-09-10

**Authors:** Jana Kottferová, Lenka Skurková, Lýdia Mesarčová, Lenka Lešková, Alena Demeová, Tomáš Jakuba

**Affiliations:** Department of Applied Ethology and Professional Ethics, University of Veterinary Medicine and Pharmacy in Košice, Komenského 73, 041 81 Košice, Slovakia; jana.kottferova@uvlf.sk (J.K.); lydia.mesarcova@uvlf.sk (L.M.); lenka.leskova@uvlf.sk (L.L.); alena.demeova@uvlf.sk (A.D.); jakubatomas@gmail.com (T.J.)

**Keywords:** egalitarian, social play, winning ratio

## Abstract

**Simple Summary:**

Social interactions within canine groups have been studied for decades. In relation to egalitarianism in social play (the “rule of 50:50”), most discussed attributes were body size or age of the play participants. We observed the social play of 14 German Shepherd puppies (two litters) at the age of 7 weeks, and once again at the age of 9 weeks (in total we observed 43 play pairs, also known as dyads). These litters were observed separately (litter 1: *n* = 28, litter 2: *n* = 15), and we evaluated 2542 interactions of social play in total. This total number included all interactions observed between the 43 dyads—1287 interactions at the age of 7 weeks old and 1255 interactions at 9 weeks old were recorded. The aim of our study was to evaluate symmetry within play interactions of puppies. Symmetry within the play has been confirmed for litter 1 at the age of 7 and 9 weeks and for litter 2 at the age of 7 weeks, but the final interpretation of our results is limited due to the small sample size and it is impossible to generalize our results for domestic dogs or a specific breed. For further studies, we would suggest an evaluation of breed differences (and including more individuals of each breed), ecological backgrounds and environmental factors regarding the development of egalitarian (or some other) play style.

**Abstract:**

The symmetry of social play in Canids has been previously studied, especially in wolves, free-ranging dogs, and within mixed-aged groups, however our study focused on symmetry and asymmetry within play interactions in two litters (14 puppies) of German Shepherd dogs (GSDs). At the age of 7 weeks, we evaluated 1287 dyadic interactions (litter 1: *n* = 339 interactions, litter 2: *n* = 948 interactions), and at the age of 9 weeks we evaluated 1255 dyadic interactions (litter 1: *n* = 433 interactions, litter 2: *n* = 822 interactions). Dyadic interactions were observed and the winning indexes were calculated for 43 pairs (dyads). The groups of puppies studied were all the same age, therefore we focused on the aspects of sex and body size as primary variables. The weight and chest circumference of all puppies were measured. The distribution of interactions showed a slight inclination to mixed-sex dyads, but we did not obtain any statistically significant results concerning the impact of body size on play interactions. Symmetry in play was observed within litter 1 at the age of 7 weeks and at the age of 9 weeks, and within litter 2 at the age of 7 weeks. Since the number of puppies in this study was too small, these results should be interpreted regarding this limitation, and cannot be generalized to a larger population of domestic dogs nor the GSD breed. In further studies, it would be interesting to compare larger samples of different breeds, under different breeding conditions, and the effect of the environment on the style of social play.

## 1. Introduction

Play can be classified into a number of categories (which are not mutually exclusive), such as social play, individual play, object play and locomotor play. Social play is defined as “a play directed at a conspecific [1,2] and in Canidae includes behaviours such as chasing and play-fighting games, mounting behaviour (i.e., mimicking copulatory behaviour) and inhibited biting [3,4]” [5]. In domestic dogs, social play is represented by actions exhibited that are also used during hunting, sexual interactions or agonistic behaviours [4,6,7], including actions unique for play (rearing up and jaw sparring). This category of play requires cooperation with play partners and therefore, we can assume that prosocial mechanisms are important for sustaining it [8]. In comparison with either object or solitary play, social play is usually performed more frequently and for a longer period in many species [3]. Hence, it can be understood as a part of the “prosocial toolkit” that needs to be rehearsed and developed in order to facilitate the establishment of longer-term social relationships [9].

A repertoire of social play in dogs expanded during domestication from unique dog–dog play into dog–human play, and studies show that social play in dogs is a marker of healthy development with a positive effect on social bonds [10,11]. However, we must remember that dog–dog play and dog–human play appear to be motivationally distinct [12] so we focused our study on the intraspecies social play.

The first evidence of social and sexual play in Canids is from 3 to 12 weeks of age, during the period of socialization [13]. Following the socialization period, the juvenile period lasts from 12 weeks to 6 months or until sexual maturity [13,14]. During this time, play continues to be common and play-partner preferences begin to form, so at the age of 6 months, there is a strong unidirectional preference for a play partner [5]. Dogs and wolves exhibit high frequencies of play behaviours even as adults [15].

The role of play in Canidae has been questioned for many decades and, currently, many hypotheses about its function exist. Play is likely to be complex and multifunctional, and therefore what appears to be an explanation of function in one species may not be applicable in another [3,16]. Firstly, the function of social play may be a reorganization of the youngsters into the adult pattern [17] and secondly, there is an adaptive function. Play has an impact both on the mental and physical traits of the individual; it can enhance the learning of social skills [18] and improve motor skills [19]. Play is also important for testing and strengthening social bonds [20,21,22,23], the development of emotional flexibility and ability to cope with unexpected situations [8]. It may be viewed as a kind of “cognitive training” [8,24] as it is a way for individuals to understand what specific communication signals mean, e.g., that biting and a specific vocalization is associated with pain [25,26]. It is also speculated that social play helps dogs develop the skills necessary for cooperation—for example, reciprocity, development of negotiation and an understanding of which individuals to trust as social partners [22]. Some studies [13,27,28] support the theory that in Canidae (both domestic and wild ones), social play has an importance for the formation of dominance relationships within litters. When puppies are raised together, they can establish their dominance hierarchy over food (competition for source), or during play without serious injuries [29].

Play has a definitive direct effect on other social interactions within the group, even if the group consists of members from different species. Some authors state that “winning or losing” games can affect the hierarchy among humans and their dogs [30]. Others contradict this by stating that play pursues the goal of “equality” [22]. It means for play to occur, both partners must win an equal proportion of play encounters [16,31,32]. This is called the 50:50 rule [32] or the egalitarian style of play. For the purpose of equal play, partners use self-handicapping (muzzle lick or voluntary laying down) or role-reversal behaviour. It is known from a previous study by Bekoff [33] that individuals who do not play symmetrically with partners may be excluded from play, therefore individuals with some advantages should express more self-handicapping or role-reversal behaviour within the play to make it more symmetrical [21,32] and to continue playing.

Contrary studies indicate that mammals initiate and play more often with individuals they can dominate [18,34]. Bauer and Smuts [35] tested the 50:50 hypothesis in adult dogs and they found, contrary to prediction, that only 5.4% of the 55 dyads tested displayed 50:50 symmetry during play, whereas 21.8% displayed complete asymmetry. Instead of egalitarian play, older animals showed significantly fewer self-handicapping behaviours than the younger animals did, and smaller dogs self-handicapped more often than larger dogs. It seems that older and larger dogs tend to be more dominant. Ward et al. [5] also tested this hypothesis within 39 dyads of dog puppies (all of whom were littermates) and they found no evidence supporting the 50:50 rule, actually finding that asymmetry increased as puppies grew older. It seems that dogs use their size, age or higher rank as an advantage over the partner. Therefore, more research is still needed to find a definitive answer for the question of how the 50:50 rule applies to Canid relationships.

The aim of our work was to contribute to the evaluation of symmetry within social play interactions in domestic canines. The second aim is to focus on the effect of sex and body size on social play in two puppy packs of German Shepherd dogs (14 puppies) and if these two variables can possibly affect the equality of play interactions and the frequencies of “winning” within the dyads of German Shepherd dogs (GSDs). We had a unique chance to observe one specific breed (German Shepherd dogs) when all the studied individuals were at the same age. It brings an opportunity to evaluate the impact of body size and sex as primary variables in social play and its symmetry (as the age effect is theoretically removed).

The environmental background of the subjects studied is also unique as the subjects did not need to compete for resources. This is unique to previous studies which were performed within groups of wolves or free-ranging dogs (where competition for limited resources influence the symmetry of play) or within mixed-aged groups (which may be a reason for asymmetry due to advantage of age and experiences). We first tested the hypothesis regarding the proportional distribution of interactions within the group in relation to the sex of individuals. We then tested the second hypothesis that a larger body size correlates to a higher probability of being the “winner” in play interactions. Finally, we investigated whether play in puppies of GSDs is egalitarian.

## 2. Materials and Methods

The study began in March and lasted until July of the same year. In April we carried out preliminary observations in order to become familiar with the study area and accustom the dogs used in the study to the presence of the observer. We also established the most visible behavioural patterns in the group of dogs and the data collection methods.

### 2.1. Study Area and Subjects of the Study

Our study was carried out in collaboration with the Slovak Police Headquarters, Department of Kynology and Hippology, where an integral part of the Department of Kynology and Hippology of the Slovak Police Headquarters is the centre for the breeding and training of police dogs. The centre is responsible for the selection of the most appropriate individuals from a genetic pool of working dogs from the German Shepherd breed, and for the breeding of selected individuals which are later used for training and service. This centre allowed us to record behavioural interactions within two litters of German Shepherd puppies (first at the age of 7 weeks and again at 9 weeks of age).

Puppy pack 1 consisted of eight puppies that were German Shepherd dogs (GSDs) (see Table 1) with an even sex distribution (4 females and 4 males). Puppy pack 2 consisted of six puppies that were GSDs (see Table 1) which also had an even sex distribution (3 females and 3 males). The total number of individuals from the combined litters (14 puppies equally divided into 7 males and 7 females) remained stable throughout the period of study. The sex of dogs was determined based on morphological characteristics. All dogs were born in the study area, so the dates of birth of each puppy was known and they were bred under the same conditions (including appropriate shelter, space for resting, quality of food and feeding regime, medical care and welfare). They were raised by professional trainers and thus spent some time in the presence of humans from birth. Details about the packs used for the purpose of our study, as well as the information about individuals, are available in Table 1.

All the animals included in our study were marked by specific dog collars (with a unique visual pattern on the collar for each individual, e.g., horizontal lines, vertical lines, dots, etc.). The filming of interactions took place in the animals’ home enclosures (indoor shelters and fenced outdoor areas).

### 2.2. Data Collection, Behavioural Observations, and Analysis

All interactions in the litter were video recorded from May until June/July and used for subsequent coding. Observations of groups were recorded twice: first within the one-week period when the puppies were 7 weeks old and when they were 9 weeks old. We obtained 4 sets of continuous records altogether, each approximately 150 to 180 h in duration. After each observation period we measured the chest circumference and weight of each puppy in both litters.

Subsequently, the records were edited so only sequences of footage containing interactions between 2 puppies remained for evaluation. Therefore, any footage that we deemed unimportant to the study, for example when puppies were asleep or during periods of no activity, were cut out. Finally, we randomly selected 10 h of footage from each of one-week period and evaluated them in terms of separate litters and ages.

The behaviour of the puppies was observed when there was no source of competition (no food, treats, toys etc.).

### 2.3. Play Observations

For the purpose of the study, selected behaviours were assessed. Behavioural patterns during dog–dog interactions were defined and characterized using indicators described by recent studies to be the indicators of play in canines [5,35]. We observed interactions defined as offensive behavioural patterns (see Table 2). Mutual interaction began after an “invitation” from one puppy to another and ended right after one of the playmates moved away or stopped the action. We did not include interactions of unsuccessful attempts ignored by the opposite partner. Only play interactions lasting for at least 60 s were included in the study.

Play interactions were coded as a second play if one of the playmates stopped playing for 20 s or more. Video recordings of the sessions were coded using Observer XT 9.0 software (Noldus Information Technologies). All videos were coded by three independent observers, J.K., T.J., and A.D. The interobserver reliability (three coders) for the play observations was 91.5%.

### 2.4. Testing of the 50:50 Ratio

According to the 50:50 rule hypothesis, the win ratio (or winning index) of most puppy dyads should be around 50:50 in the context of social play [20,22]. To test the equality in the context of social play, a relative winning index was calculated for each individual within each dyad.

In the context of play, offensive behaviours were considered “winning” behaviours, while submissive or self-handicapping behaviours were considered “losing” behaviours (the same terms were used also in other studies on play in dogs or wolves [5,35,36]). In this way, it was possible to calculate the ratio for each dyad. The proportion of winning interactions ranged from 50:50 (complete symmetry of a play) to 100:0 (complete asymmetry).

(1) $$Winning\;Index=\frac{A\;\mathrm{offensive}\;-\;B\;\mathrm{offensive}}{A\;\mathrm{offensive}\;+\;B\;\mathrm{offensive}}$$

Subsequently, we analysed how size or sex of the partners within the dyads influenced play behaviour.

## 3. Results

### 3.1. Play Interactions within the Litter in Relation to the Sex of Puppies

Data obtained from our observations are presented for each litter (puppy pack) separately at the age of 7 weeks and the age of 9 weeks. We observed 28 dyads in puppy pack 1 and 15 dyads in puppy pack 2. Therefore, 43 dyadic interactions were observed altogether throughout the study. The total number of interactions observed was 2542 (see Table 3). At 7 weeks of age we observed 1287 dyadic interactions within both puppy packs and at 9 weeks of age, we observed 1255 dyadic interactions in both puppy packs.

We tested the hypotheses discussing whether the distribution of interactions between sexes was proportional within the puppy packs. In both puppy packs, the proportion of sexes was equal (50:50) (see Table 1). In puppy pack 1, any individual of any sex had an opportunity to interact with the individual of same sex in three out of seven cases (43%) or with the individual of opposite sex in four out of seven cases (57%). In puppy pack 2, any of the puppies has an opportunity to interact with the individual of same sex in two out of five cases (40%) or with the individual of opposite sex in three out of five (60%) of cases. The total numbers of interactions observed within each puppy pack are presented in Table 3. Results (presented in Table 3) were statistically analysed with a χ^2^ test for goodness of fit at the significance level of alpha < 0.05 (*) (Table 4). We found that both males and females preferred a partner of the opposite sex slightly more than expected. These results were the same in both puppy packs and at the age of 7 weeks and the age of 9 weeks (Table 4).

### 3.2. Dyadic Play Interactions within the Litter in Relation to Size (Weight vs. Chest Circumference) of Puppies

Data on the individuals in both packs are in Table 1. To evaluate the effect of the body size of puppies on dyadic interactions, we measured the chest circumference and weight of the puppies. To decide which of the two given variables were going to be used for the purpose of evaluation, we analysed the correlation between the weight and chest circumference of the puppies in puppy pack 1 and puppy pack 2. The results were analysed statistically and Pearson’s correlation coefficient was calculated. A moderate to strong positive correlation was found between the two variables (weight and chest circumference) at the level of significance *p* < 0.05 (*).

Based on the previous results (correlation of the two variables), we decided to use only weight data to test the hypothesis that puppy size and winning during interactions are positively correlated. Winning indices were calculated for each dyad and both individuals interacting in these dyads (this was calculated separately for pack 1 (Table 5) and pack 2 (Table 6)), as was the mean value, median and standard deviation.

A winning index of 0 would represent complete symmetry, with each partner being in the winning position an equal amount of times. A winning index of −1 or 1 represents complete asymmetry, with one individual being in the winning position the entire time the dyad was seen playing [36].

**Figure 1:**
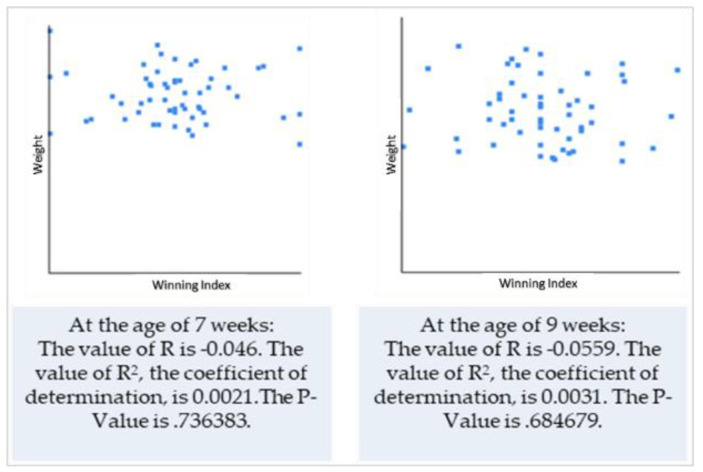
Correlation between the weight of puppies and winning indices—puppy pack 1.

**Figure 2:**
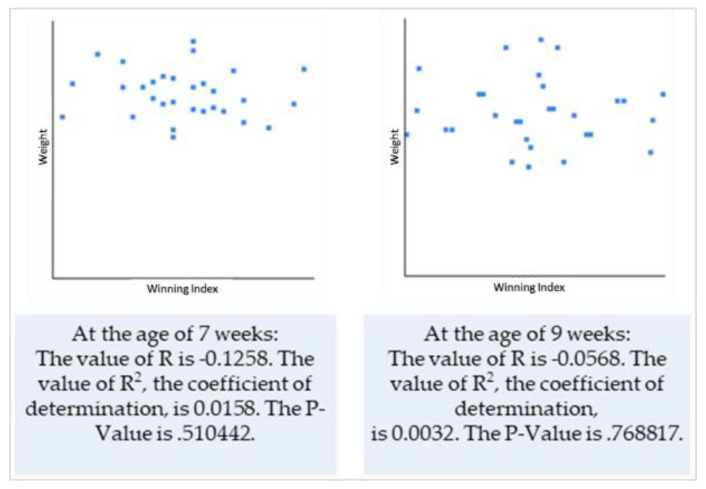
Correlation between the weight of puppies and winning indices—puppy pack 2.

When testing the hypothesis regarding the correlation between the winning index and puppy’s body size and when calculating the Pearson’s correlation coefficient, we found that although there is technically a negative correlation between the two variables, the relationship between variables was only weak and the results were not significant at *p* < 0.05 (Figure 1 and Figure 2).

### 3.3. Testing the 50:50 Rule

Firstly, we identified individuals winning and losing in each interaction within the dyad. Next, we calculated the winning indexes for each individual in a dyad. We also took into account the number of wins of the opposite partner, but did not consider the occurrence of self-handicapping behaviours or role-reversal behaviours.

The 50:50 rule means that the number of wins within the dyad should be the same for both partners. Then the style of play within the dyad could be considered as symmetric or equalitarian. A 50:50 distribution is represented when the value of winning index = 0 and a 40:60 distribution of wins is represented with the values 0.20 (or −0.20).

We tested the 50:50 rule in relation to the age of puppies (7 weeks vs. 9 weeks) and in relation to the combination of play partners’ sexes within the dyads (female–female dyads vs. male–male dyads vs. mixed-sex dyads). The total number of dyads tested was 43.

In relation to the age of puppies, at the age of 7 weeks a distribution of 50:50 (complete symmetry) was observed in 6.98% of dyads and a distribution from 50:50 to 60:40 was observed in 58.14% of dyads. At the age of 9 weeks, a distribution of 50:50 was observed within 11.63% of dyads and the distribution of 50:50–60:40 was observed within 48.84%. Winning indexes represented the degree of asymmetry (or symmetry) within each dyad and were used to test the distribution of that data against the normal distribution. Overall data in our study did not differ from the normal distribution (Kolmogorov–Smirnov test of normality: D = 0.10968, *n* = 86, *p* = 0.23441, SD = 0.333617) as well as data for male–male dyads or female–female dyads (not different from the normal distribution).

To test symmetry in play, the Wilcoxon signed rank test was used (WI = 0) for each age group separately. Different results were confirmed for 7- and 9-week old groups. In the younger group, symmetry was confirmed (sum of signed ranks W = 93, 9 = 0.5470, *n* = 43), but in the older group it was not (W = 370, 9 = 0.0063, *n* = 43). When testing the symmetry of each litter at different ages (4 groups), symmetry was confirmed for litter 1 at the age of 7 weeks (W = 22, *p* = 0.7762, *n* = 28) and at the age of 9 weeks (W = 80, *p* = 0.2310, *n* = 28). It was also confirmed for litter 2 at the age of 7 weeks (W = 22, *p* = 0.5614, *n* = 15). The symmetry of play was not confirmed for puppies in litter 2 at the age of 9 weeks (W = 98, *p* = 0.0034, *n* = 15).

Taking into account the fact that these two age groups must be treated as repeated measures, a Wilcoxon matched-pairs signed rank test was used to test the difference between them (W = 365, *p* = 0.0267, *n* = 43). A significant difference was confirmed with the median of differences 0.06 (95% CI: (−0.02 to 0.16)). The Spearman coefficient confirmed the effect of pairing as it was significant and reached a value of 0.5735 with *p* < 0.0001. Statistical analyses were conducted for the first litter with the following results: (W = 82, *p* = 0.3588, *n* = 28), median of differences was 0.06 (−0.02 to 0.16), Spearman coefficient was significant and reached a value of 0.5735 with *p* < 0.0001. Significant differences between age groups were not confirmed. Results of analyses for the second litter: (W = 83, *p* = 0.0157, *n* = 15), median of differences was 0.18 (−0.13 to 0.32), Spearman coefficient did not confirm effecting of pairing, it was not significant −0.1321 with *p* = 0.3195. Significant differences between age groups were confirmed, but the nonsignificant Spearman coefficient means that the effect of pairing was not significant.

**Figure 3:**
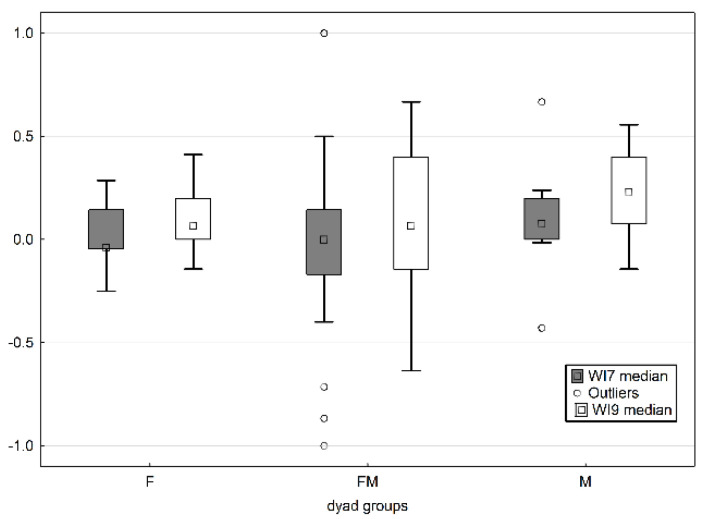
Box-plot graph of winning indices for 7 weeks vs. 9 weeks (WI7 vs. WI9) old dyads with respect to combination of play partners’ sexes within the dyads (F: female–female dyads vs. M: male–male dyads vs. FM: mixed-sex dyads).

In relation to combination of play partners’ sexes within the dyads, the testing with the Kruskal–Wallis ANOVA was used. The box-plot graph of winning indexes (WIs) for sexually different (F, M, FM) groups of dyads in both age pseudoreplicates shows that the WIs oscillate more or less about 0 (Figure 3).

Significant differences were not confirmed for the younger group (Kruskal–Wallis test: H (2, *n* = 43) = 1.2046 *p* = 0.5475) nor for the older group (H (2, *n* = 43) = 1.9808 *p* = 0.3714). Significant differences for symmetry in relation to the sex composition of dyads were not confirmed for litter 1 nor litter 2 (tested separately), but we assume that nonsignificance is likely to be due to the low sample size.

## 4. Discussion

### 4.1. Play Interactions within the Litter in Relation to the Sex of Puppies

Social play can be directed towards both sexes within the group, but some differences have been previously observed [5,35,37]. In relation to the initiation of play, according some authors [35] females interact more than males but it has been described that males initiate more interactions than females [5,37,38]. Results on preferences for play partners in relation to sex are still ambiguous.

In our study, the distribution of dyadic interactions within the litter was slightly more directed towards the mixed-sex dyads than expected. We found that both males and females preferred a partner of the opposite sex. Such a result has been described also in the study of Bauer and Smuts [35], according to which males were not willing to play with other males. On the other hand, Ward et al. [5], and Lund and Vestergaard [37] described no sex preference of males for a play partner, but in females a preference for females as a play partner was observed [35]. The study conducted by Pal [38] supports our results, because they found in a group of free-ranging dogs (*n* = 24) that male puppies initiate more interactions with females (and females more often with males). A possible explanation for such a preference of an opposite sex partner may be that intersexual interactions improve the social competence of the individual to communicate and interact with the opposite sex partner at the reproductive age (training function of play).

### 4.2. Dyadic Play Interactions within the Litter in Relation to Size (Weight vs. Chest Circumference) of Puppies

From testing the hypothesis on the correlation between the winning index and puppy’s body size and by calculating the Pearson’s correlation coefficient, we found that, although there is technically a negative correlation between two variables, the relationship between variables was only weak and the results were not significant at *p* < 0.05. Therefore, our hypothesis that there is a correlation between the body size of puppies and their “success” within social play was not confirmed. These results are similar to those according to Bauer and Smuts [35]. Their study showed that relative size did not reveal a robust effect on any aspect of play style. It is contradictory to other researchers [5] which argue that dogs use their size as an advantage and larger individuals exhibit a tendency to behave more dominantly. We did not observe such tendencies and so we can only speculate that at such a young age, not only size but the progression in the development of motor skills, and probably some other individual factors, may play a more important role to be a “winner” than body size. Similarly to these speculations, a study on packs of free-ranging dogs [39] revealed that age was a better predictor of dominance than body size. Therefore, we can only assume that age would also be a better indicator for winning within the play context. Although it seems that body size is probably not important for the probability of winning during the play, our results were statistically not significant, hence we cannot make any relevant conclusions. More importantly, one of the limitations of the interpretation of these results includes the fact that individuals in our study were at the same age, were the same breed and were within the same litter. The relative difference in body size between the puppies may not be large enough to have a significant influence on the play success. This aspect of our study may be an aim of future investigations.

### 4.3. Testing the 50:50 Rule

We tested puppies at the age of 7 weeks and 9 weeks and we obtained mixed results. The hypothesis regarding the symmetry of social play within two puppy packs of German Shepherd dog was confirmed at the age of 7 weeks for both litters and at the age of 9 weeks for litter 1. However, the low sample size does not provide enough data to make any definitive conclusions and further analysis with more individuals is recommended. In relation to the sex composition of dyads, significant differences have not been confirmed (for the younger nor older group), but nonsignificance is very likely to be due to the low sample size. In previous studies, wolves or free-ranging dogs, or dogs from different age categories had been studied, but these Canid categories had different ecological settings from the ones our puppies had. Therefore, when discussing differences, we should take into account at least two possible variables (age and environmental conditions of groups studied) that may influence the results. One study [35] showed that the 50:50 rule is not present within mixed-age dyads of domestic dogs, as in 20.8% of dyads the distribution of wins and losses varied from 50:50 to 60:40 [35]. An explanation of such a difference (in comparison with our results) can be based on the simple fact that they studied mixed-age groups and we studied social play in dyads of littermates, therefore age was the same for all play partners.

In wolves, Essler et al. [36] revealed that the tendency for symmetry in play is more often presented in youngsters (than in adults). Dyads consisting of two puppies had significantly more equal play than dyads consisting of one puppy and one adult.

Interesting results were found also in a study on dog puppies (result: play style is not following the 50:50 rule within a play context) [5]. Domestic dog puppies decrease their symmetry in play over time when comparing the time periods between 3–8 and 10–23 weeks of age. No such a decrease can be detected when comparing the time periods of 10–23 and 27–40 weeks of age [5]. In the previously mentioned study of Essler et al. [36], such a decrease was not observed (the animals used for this study were 12–20 weeks old). They speculated that wolf puppies establish their dominant relationships later than dogs and therefore play remains more symmetrical for longer periods. An explanation for differences in the egalitarian play style of wolves and dogs is due to their differences in dependence; while wolves are highly reliant on others in the pack, dogs have reduced pack involvement in raising pups [40,41,42,43].

One of the possible explanations for more equalitarian play in puppies in relation to age may be that at age 7 or 9 weeks a dominance hierarchy is not established. Competitive behaviour in a playful context usually appears at the age of 3 or 4 weeks and if puppies remain in the same group (litter), early established relationships within dyads become stable at 11 weeks of age [13]. Before the establishment of hierarchy, according to Serpell [44] and Wright [45], considerable instability has been demonstrated in the dyadic relationships between littermates. Citing Wright “far more than might be expected to underlie a straightforward progression towards a stable hierarchy” [45]. The social hierarchy should be established by 15 weeks of age in 88% of the puppies [13,14]. Therefore, at the age 7 (or 9) weeks the “training” function of play may be still more important than the “hierarchy-establishment” function.

Another explanation for the differences in results can be the environment and availability of sources. It means that the food, shelter, etc., in our study was provided for the puppies by humans, and therefore there was no need for existential competition for sources and play may show more symmetry.

Although, the sample size in this study was too small, therefore our findings cannot be generalized to the larger population of German Shepherd dogs and further investigation is needed. Few studies about the steepness in captive groups of various purebred and mixed breed dogs are suggestive of a tolerant [46] or even egalitarian dominance style [47]. These studies indicate that breeds vary greatly in their genetic predisposition to aggression. It would also be interesting to continue in the study of the style of social play with regard to breed differences.

In some studies on purebred dogs [48,49], puppies that are separated early from their mothers and then raised by humans were described as aggressive. It seems that also early weaning is a factor contributing to the development of increased aggression and resource guarding in dogs [50]. These factors (breed and early separation) may interact together and create a more aggressive phenotype.

Bonanni et al. [39] hypothesized that dogs under strong artificial selection may exhibit a more despotic dominance style than free-ranging dogs. According to Bonanni et al. [39], dogs may be more cooperative than is usually supposed and based on their results, contradicted the view that domestication has increased despotism in dogs.

Therefore, studies focused on breed differences or the age of separation from the mother and environmental status of dogs (free-ranging, pets, working dogs or wild dogs) and the impact these factors have on social play style would be a subject for further study on the social interactions within the social play of *Canis familiaris*.

## 5. Conclusions

Social play has a definitive direct effect on other social interactions within the group. We had a unique chance to observe one specific breed (German Shepherd dogs) when all the studied individuals were at the same age which means opportunity to evaluate the impact of body size and sex as primary variables in social play and its symmetry (as the age effect is theoretically removed). We found that both males and females preferred a partner of the opposite sex. Our hypothesis that there is a correlation between the body size of puppies and their “success” within social play was not confirmed. The hypothesis regarding the symmetry of social play within two puppy packs of German Shepherd dog was confirmed at the age of 7 weeks for both litters and at the age of 9 weeks for litter 1. However, the low sample size does not provide enough data to make any definitive conclusions and further analysis with more individuals is recommended.

## Figures and Tables

**Table 1:** Data on the individuals included in our study—14 puppies of German Shepherd Dogs (GSDs) from two litters (A–H puppies are from litter 1, I–N puppies are from litter 2).

Litter	Birth	Dog ID	Sex	Weight (kg)		Chest Circumference (cm)	
	(month)			7 weeks	9 weeks	7 weeks	9 weeks
1	April	A	Female	4.1	5.6	38	45
		B	Female	4.9	7.0	39	43
		C	Male	4.5	5.4	39	46
		D	Male	5.2	7.1	42	47
		E	Female	3.8	5.0	36	40
		F	Male	4.2	5.1	37	40
		G	Male	4.3	6.4	39	42
		H	Female	4.5	6.7	38	46
2	March	I	Female	5.5	6.0	47	49
		J	Female	5.2	5.1	47	47
		K	Male	5.9	5.3	45	47
		L	Male	6.7	7.5	50	51
		M	Female	5.7	5.3	42	46
		N	Male	5.4	5.8	45	48

**Table 2:** Ethogram of behaviours displayed in the play context in domestic dogs (*Canis familiaris*) adapted from previous study [35]. W—winner, L—loser.

Behaviour (Attacks/Pursuits)	Definition
Forced down	W uses sufficient physical force to cause L to drop partially or completely to the ground; force may be applied with a bite, push/tackle, body slam or some other forceful movement.
Chin over	W places chin over L’s back, usually right behind the neck or near L’s shoulders, W’s chin may or may not touch L but W’s chin must be at or near a 90-degree angle in relation to the plane of L’s spine.
Mount	W rears up to place forelegs on L’s back in a front lateral or rear mount position, differentiated from push/tackles by W’s rounded spine position during the mount and distinct pause in the stable mount position, which may or may not be accompanied by thrusting.
Over	W sits on, stands over, or lies over L at least 25% of W’s torso over L torso.
Overs during downs	W stands over or lies on L with at least 50% of W’s torso over L’s torso (or vice versa: 50% of L’s torso is under W’s torso), or W sits and exerts weight directly on L’s head or torso with a distinct pause in the sitting position.
Paw on	W stands up on its hind legs and puts front legs on R’s shoulders, usually silent and with open mouth, individuals can bite each other.
Push/tackle	W rears up and places one or both front paws on L with forceful contact; forelimbs may or may not be wrapped around L’s body in a tackle grasp (if interaction results in a down, it is coded as forced down, instead of independent push/tackle).

**Table 3:** Numbers of intrasexual and intersexual interactions in dyads in 7th and 9th week.

Number of Interactions in Dyads								
Litter	Week	Total	Male vs. Male		Male vs. Female		Female vs. Female	
pack			number	%	number	%	number	%
1	7	339	61	18.00%	210	61.94%	68	20.05%
	9	433	75	17.30%	261	60.27%	97	22.40%
2	7	948	175	18.40%	564	59.49%	209	22.05%
	9	822	168	20.44%	481	58.52%	173	21.05%

**Table 4:** Interactions in relation to the sex of the individuals (comparison of the same sex and the opposite sex interactions: expected vs. observed proportions or frequencies).

**Both puppy Packs**	**Sex**	**Observed (No.)**	**Expected (No.)**	**Difference**	***x*^2^ Value**	***p*-Value**	**alpha < 0.05**
All interactions (*n* = 2542)	Same	926	997	−71.00	8.545	0.00347	* significant
	Opposite	1616	1545	71.00			
**Puppy pack 1**	**Sex**	**Observed (%)**	**Expected (%)**	**Difference**	***x*^2^ value**	***p*-value**	**alpha < 0.05**
Interaction of females	Same	26	43	−17.00	11.791	0.0006	* significant
	Opposite	74	57	17.00			
Interaction of males	Same	22	43	−21.00	17.993	0.00002	* significant
	Opposite	78	57	21.00			
**Puppy pack 2**	**Sex**	**Observed (%)**	**Expected (%)**	**Difference**	***x*^2^ value**	***p*-value**	**alpha < 0.05**
Interaction of females	Same	27	40	−13.00	7.042	0.00796	* significant
	Opposite	73	60	13.00			
Interaction of males	Same	25	40	−15.00	9.375	0.0022	* significant
	Opposite	75	60	15.00			

* The significance level of alpha < 0.05.

**Table 5:** Winning index calculated for each individual/each dyad (puppy pack 1). Winning index of 0 represents complete symmetry (each partner within dyad was in the position of a winner an equal amount of times).

Puppy Pack 1—Winning Indexes for Dyadic Play Interactions											
**7th Week of age**											
**Puppy**	**A** **♀**	**B** **♀**	**C** **♂**	**D** **♂**	**E** **♀**	**F** **♂**	**G** **♂**	**H** **♀**	**WI (Mean)**	**Median**	**SD**
**A** **♀**	*	0.167	−0.23	1	0	0.2	−0.06	0.29	0.1952857	0.167	0.3961341
**B** **♀**	−0.167	*	0	−0.19	0.14	−0.71	0.14	−0.04	−0.1181429	−0.04	0.2918861
**C** **♂**	0.23	0	*	0.67	−0.13	0.2	−0.43	0.5	0.1485714	0.2	0.3739621
**D** **♂**	−1	0.19	−0.67	*	1	0.11	0	−0.4	−0.11	0	0.6547773
**E** **♀**	0	−0.14	0.13	−1	*	−1	−0.87	−0.25	−0.4471429	−0.25	0.4926701
**F** **♂**	−0.2	0.71	−0.2	−0.11	1	*	0.08	−0.08	0.1714286	−0.08	0.4836124
**G** **♂**	0.06	−0.14	0.43	0	0.87	−0.08	*	0.27	0.2014286	0.06	0.3559695
**H** **♀**	−0.29	0.04	−0.5	0.4	0.25	0.08	−0.27	*	−0.0414286	0.04	0.322874
**9th Week of age**											
**Puppy**	**A** **♀**	**B** **♀**	**C** **♂**	**D** **♂**	**E** **♀**	**F** **♂**	**G** **♂**	**H** **♀**	**WI (Mean)**	**Median**	**SD**
**A** **♀**	*	0	−0.25	0.67	0.2	0	0.1	0.41	0.1614286	0.1	0.3019618
**B** **♀**	0	*	0.11	−0.14	0.07	0	0.2	0	0.0342857	0	0.1067485
**C** **♂**	0.25	−0.11	*	0.4	−0.18	0.25	−0.14	0.4	0.1242857	0.25	0.2585122
**D** **♂**	−0.67	0.14	−0.4	*	0.4	0.06	0.08	0	−0.0557143	0.06	0.3598081
**E** **♀**	−0.2	−0.07	0.18	−0.4	*	−0.64	−0.55	−0.14	−0.26	−0.2	0.2874601
**F** **♂**	0	0	−0.25	-	0.64	*	0.23	−0.16	0.0766667	0	0.32104
**G** **♂**	−0.1	−0.2	0.14	−0.08	0.55	−0.23	*	0.14	0.0314286	−0.08	0.2720557
**H** **♀**	−0.41	0	0.4	0	0.14	0.16	−0.14	*	0.0214286	0	0.2548482

* The level of significance *p* < 0.05.

**Table 6:** Winning index calculated for each individual/each dyad (puppy pack 2). Winning index of 0 represents complete symmetry (each partner within dyad was in the position of a winner an equal amount of times).

Puppy Pack 2—Winning Indexes for Dyadic Play Interactions									
**7th Week of age**									
**Puppy**	**I** **♀**	**J** **♀**	**K** **♂**	**L** **♂**	**M** **♀**	**N** **♂**	**WI (Mean)**	**Median**	**SD**
**I** **♀**	*	−0.04	−0.06	−0.17	−0.08	−0.06	−0.082	−0.06	0.0511859
**J** **♀**	0.04	*	0.1	0.02	−0.04	0.02	0.028	0.02	0.0501996
**K** **♂**	0.06	−0.1	*	0.24	0.12	0.02	0.068	0.06	0.1253794
**L** **♂**	0.17	−0.02	−0.24	*	0.12	−0.02	0.002	−0.02	0.1594365
**M** **♀**	0.08	0.04	−0.12	−0.12	*	0.22	0.02	0.04	0.1442221
**N** **♂**	0.06	−0.02	−0.02	0.02	−0.22	*	−0.036	−0.02	0.108074
**9th Week of age**									
**Puppy**	**I** **♀**	**J** **♀**	**K** **♂**	**L** **♂**	**M** **♀**	**N** **♂**	**WI (Mean)**	**Median**	**SD**
**I** **♀**	*	−0.04	0.25	0.02	0.27	0.57	0.214	0.25	0.241516
**J** **♀**	0.04	*	0.07	0.03	0.09	0.62	0.17	0.07	0.2526856
**K** **♂**	−0.25	−0.07	*	0.11	−0.19	0.43	0.006	−0.07	0.2740073
**L** **♂**	−0.02	−0.03	−0.11	*	0.14	0.56	0.108	−0.02	0.2684586
**M** **♀**	−0.27	−0.09	0.19	−0.14	*	0.4	0.018	−0.09	0.2716063
**N** **♂**	−0.57	−0.62	−0.43	−0.56	−0.4	*	−0.516	−0.56	0.095551

* The level of significance *p* < 0.05.
